# Virtual and in-person postpartum visit attendance, emergency department use, and hospital readmissions before, during, and after the COVID-19 pandemic: a retrospective cohort study in a diverse urban Bronx population

**DOI:** 10.21203/rs.3.rs-10607107/v1

**Published:** 2026-09-11

**Authors:** Rachel Nordlicht, Jessica Atrio, Kavita Vani

**Affiliations:** Albert Einstein College of Medicine; Alice L. Walton School of Medicine; Albert Einstein College of Medicine and Montefiore Medical Center

**Keywords:** Postpartum care, COVID-19 pandemic, Maternal health disparities, Telehealth, Urban safety-net population, Acute care utilization

## Abstract

**Objective:**

To evaluate postpartum in person and telehealth follow-up attendance before, during, and after the COVID-19 pandemic in a large, urban, racially diverse obstetric population.

**Methods:**

We conducted a retrospective cohort study of 13,874 patients who received prenatal care and delivered at Montefiore Medical Center between June 2018 - May 2022. Four periods were defined based on key COVID-19 milestones: pre-COVID (before 3/11/2020, pandemic declaration), early COVID (3/11/2020–6/11/2020, elective procedures cancelled), late COVID (6/12/2020–6/15/2021, elective procedures resumed, no widespread vaccination), and post-COVID (after 6/15/2021, vaccines widely available). The primary outcome was postpartum visit (PPV) attendance within 12 weeks of delivery. Chi-square tests compared PPV attendance across periods. Logistic regression adjusted for demographics, prenatal care, comorbidities, delivery outcomes, and NICU admission. Secondary outcomes included emergency room (ER) visits and readmissions within 12 weeks.

**Results:**

There were 6,989 (50.4%) deliveries pre-COVID, 944 (6.8%) during early COVID, 3,273 (23.6%) during late COVID and 2,668 (19.2%) post-COVID. Compared to pre-COVID PPV attendance of 46.2%, PPV rates increased significantly during early (73.5%), late (63.7%) and post-COVID (59.8%), p < 0.01). After adjustment, compared to pre-COVID, patients who delivered during early COVID had a 3.5x higher odds of attending a PPV (aOR 3.50, 95% CI 2.97–4.11), 1.98x higher odds during late COVID (aOR 1.98, 95% CI 1.80–2.17) and 1.6x higher odds post-COVID (aOR 1.61, 95% CI 1.45–1.78), all p < 0.01. Compared to pre-COVID, ER visits were less likely during early (aOR 0.61, 95% CI 0.49–0.76, p < 0.01) and late COVID (aOR 0.87, 95% CI 0.77–0.99, p = 0.03), and hospital readmission were more likely post-COVID (OR 1.35, 95% CI 1.07–1.69, p = 0.010).

**Conclusion:**

PPV attendance increased during the COVID-19 pandemic and remained elevated post-pandemic. This pattern may reflect forced changes in care delivery, such as expanded telehealth use, that may have helped reduce longstanding barriers to follow-up. Sustained implementation of flexible care models may help preserve these gains.

## Introduction

The postpartum period is a critical window for identifying and addressing acute complications, supporting recovery from pregnancy, and establishing long-term preventive care.^1^ The American College of Obstetricians and Gynecologists (ACOG) recommends timely and ongoing postpartum follow-up, yet attendance at postpartum visits in the United States remains suboptimal, particularly among individuals facing structural and socioeconomic barriers to care.^1–3^ Missed postpartum care represents a lost opportunity to mitigate severe maternal morbidity and mortality, the majority of which occur after delivery and are often preventable.^4–6^ Improving engagement in postpartum care is therefore a central strategy in efforts to reduce maternal health disparities.

The COVID-19 pandemic disrupted obstetric care delivery beginning in March 2020. Health systems implemented changes including reduced in-person visits, shortened postpartum hospital stays, and expanded telehealth services.^7–9^ Concurrently, perinatal mental health burden increased, in addition to disruptions in postpartum depression screening and contraceptive access.^10–11^ These system-level changes may have altered patterns of postpartum health care utilization, including attendance at scheduled visits and reliance on acute care services, particularly in racially and ethnically diverse, urban populations disproportionately affected by both COVID-19 and maternal morbidity.^12–14^

This study sought to evaluate postpartum care utilization – including in person and telehealth postpartum visit attendance, ER visits, and hospital readmissions – before, during, and after the height of COVID-19 pandemic in 2020 in a large, urban, academic health care system. We compared outcomes among individuals delivering before the pandemic, during the early and later phases of the pandemic in 2020–2021, and after widespread vaccine availability, using these time periods as markers of evolving healthcare delivery and access during the pandemic.

## Methods

After Institutional Review Board approval with a waiver of informed consent, we conducted a retrospective cohort study of all pregnant persons delivering at Montefiore Medical Center (MMC), a large, urban, academic tertiary care center serving a racially and ethnically diverse population with two labor and delivery campuses. The study period spanned from June 1, 2018 to May 31, 2022. A structured query of the electronic medical record (EMR) to extract maternal, pregnancy, delivery, and infant data from standardized fields was conducted.

Persons were included if they received prenatal care within MMC and delivered between 24 and 42 weeks’ gestation during the study period. Receipt of prenatal care was defined as more than one documented prenatal visit in an Obstetric or Family Medicine clinic. This criterion was used to exclude individuals who obtained prenatal care primarily in affiliated community-based clinics and were expected to return their outside clinic for postpartum care. Persons were excluded if prenatal care occurred at another institution or if delivery occurred before 24 weeks’ gestation. For individuals with more than one delivery during the study period, only the first pregnancy was included to avoid correlated observations.

The primary exposure was timing of delivery relative to the COVID-19 pandemic. Deliveries were categorized into four distinct periods defined by key institutional and public health milestones. The pre-COVID period included deliveries occurring before March 11, 2020, prior to the World Health Organization’s declaration of COVID-19 as a global pandemic. The early COVID period spanned March 11, 2020 through June 11, 2020, corresponding to the initial surge in New York City and cancellation of elective procedures within the health system. The late COVID period extended from June 12, 2020 through June 15, 2021, when elective procedures had resumed but vaccines were not yet widely available to the general public. The post-COVID period included deliveries occurring after June 15, 2021, when COVID-19 vaccines became widely accessible in New York State.^15^

The primary outcome was attendance at a postpartum visit (PPV), defined as any completed outpatient visit with MMC within 12 weeks of delivery. PPV attendance was analyzed as a binary variable (attended vs. not attended). Postpartum visits were also assessed at individual timepoints across the 12 weeks at intervals of 0-<3 weeks, 3-<9 weeks, and 9–12 weeks. Telehealth was rapidly implemented after declaration of the COVID-19 pandemic in our health system; however, we do not have visit type data available within our data set. As a result, we are unable to distinguish between whether visits attended occurred virtually or in-person. Secondary outcomes included emergency room (ER) visits and hospital readmissions occurring within 12 weeks of delivery.

Maternal characteristics included age at delivery, race, ethnicity, parity, insurance status, number of prenatal visits, gestational age at initiation of prenatal care, multifetal gestation, preterm delivery (< 37 weeks), and mode of delivery. Maternal medical comorbidities were identified using ICD-9 and ICD-10 diagnosis codes and included hypertensive disorders of pregnancy (chronic hypertension, gestational hypertension, preeclampsia with or without severe features, eclampsia, HELLP syndrome), diabetes (pregestational and gestational), obesity, and preexisting mental health conditions (including depression, bipolar disorder, and schizophrenia). Infant characteristics included gestational age at delivery, neonatal intensive care unit (NICU) admission, stillbirth and infant length of hospitalization. In the Montefiore system, all neonates born before 34 weeks’ gestation are routinely admitted to the NICU. Stillbirth was defined as Apgar scores of 0 at both 1 and 5 minutes and/or the presence of an ICD-9 or ICD-10 diagnosis code indicating intrauterine fetal demise or stillbirth.

Participant characteristics were summarized using descriptive statistics. Categorical variables were compared across COVID-19 time periods using chi-square tests or Fisher’s exact tests, as appropriate. Continuous variables were assessed using one-way ANOVA or Kruskal–Wallis tests based on distribution. Unadjusted PPV attendance rates were compared across pandemic periods using chisquare analysis. Multivariable logistic regression models were constructed to evaluate the association between delivery during each COVID-19 period and PPV attendance, using the pre-COVID period as the reference group. Adjusted odds ratios (aORs) and 95% confidence intervals (CIs) were calculated. Covariates were selected a priori based on clinical relevance and known associations with postpartum care engagement and included maternal age, race, ethnicity, insurance status, parity, number of prenatal visits, gestational age at initiation of care, maternal medical comorbidities, mode of delivery, preterm delivery status, multifetal gestation, gestational age at delivery, NICU admission, infant length of hospitalization, and stillbirth.

Separate multivariable logistic regression models were constructed to assess associations between pandemic period and secondary outcomes (ER visits and hospital readmissions). These models adjusted for the same covariates. Records with missing exposure, outcome, or covariate data were excluded from regression analyses. Missingness was < 5% for all variables. A two-sided alpha of 0.05 was used to determine statistical significance. All analyses were performed using Stata version 18.0 (StataCorp LLC, College Station, TX).

## Results

Among the 17,711 people who delivered at MMC during the study time periods, 13,874 (78.3%) received prenatal care within MMC and delivered between 24–42 weeks. Of these, 6,989 (50.4%) delivered during the pre-COVID period, 944 (6.8%) during early COVID, 3,273 (23.6%) during late COVID, and 2,668 (19.2%) during the post-COVID period.

Maternal demographic and obstetric characteristics throughout COVID-19 time periods are demonstrated in Table 1. More than 75% of patients were insured through Medicaid and approximately 30% identified as Black and over 40% as Hispanic across time periods. Of note, cesarean delivery rates increased steadily across periods, rising from 33.5% pre-COVID to 40.0% post-COVID (p < 0.001). The prevalence of gestational diabetes increased substantially over time (9.5% pre-COVID vs. 17.3% post-COVID, p < 0.001), while hypertensive disorders of pregnancy peaked during late COVID (30.3%) and were lowest in the post-COVID period (18.7%, p < 0.001).

Postpartum visit (PPV) attendance within 12 weeks differed significantly by pandemic period (p < 0.001) (Table 2). During the pre-COVID period, 46.2% of patients attended a PPV. Attendance increased markedly during early COVID to 73.5%, remained elevated during late COVID at 63.7%, and was 59.8% during the post-COVID period. Additionally, increased attendance during pandemic periods was observed across all postpartum time intervals. Attendance within 0–3 weeks nearly doubled during early COVID compared to pre-COVID (47.9% vs. 24.4%, p < 0.001). Similar increases were observed at 3–8 weeks (46.9% vs. 21.7%, p < 0.001) and 9–12 weeks (22.0% vs. 17.5%, p = 0.001). Although attendance declined slightly from early to late and post-COVID periods, rates remained significantly higher than pre-pandemic levels across all time points.

In unadjusted analyses, delivery during pandemic periods was strongly associated with increased odds of PPV attendance compared to the pre-COVID period (Table 3). In logistic regression models adjusting for maternal demographics, prenatal care utilization, comorbidities, delivery characteristics, and NICU admission, these associations remained significant (Table 4). Compared to the pre-COVID period, patients delivering during early COVID had 3.5-fold higher odds of attending a PPV (aOR 3.50, 95% CI 2.97–4.11), those delivering during late COVID had 1.98-fold higher odds (aOR 1.98, 95% CI 1.80–2.17), and those delivering post-COVID had 1.61-fold higher odds (aOR 1.61, 95% CI 1.45–1.78), all p < 0.001. Other factors independently associated with higher postpartum attendance included cesarean delivery, more prenatal visits, nulliparity, preterm delivery, and select maternal comorbidities such as hypertensive disorders of pregnancy and prior mental health diagnoses. Conversely, multiple gestation pregnancies and gestational diabetes were associated with lower odds of postpartum follow-up (Table 4, all p < 0.01).

Emergency room visits within 12 weeks postpartum were less likely during pandemic periods compared to pre-COVID (Table 5). In adjusted analyses, delivery during early COVID was associated with a 39% reduction in odds of ER utilization (aOR 0.61, 95% CI 0.49–0.76, p < 0.001), and delivery during late COVID was associated with a 13% reduction (aOR 0.87, 95% CI 0.77–0.99, p = 0.028). No significant differences in ER utilization were observed during the post-COVID period relative to pre-COVID (aOR 0.91, 95% CI 0.80–1.04, p = 0.177). Hospital readmission within 12 weeks postpartum did not differ significantly during early (aOR 1.31, 95% CI 0.95–1.81, p = 0.100) or late COVID (aOR 1.17, 95% CI 0.95–1.45, p = 0.143) compared to pre-COVID. However, delivery during the post-COVID period was associated with a 35% increase in odds of hospital readmission compared to pre-COVID (aOR 1.35, 95% CI 1.07–1.69, p = 0.010).

## Discussion

### Principal Findings:

In this large, diverse, urban cohort, postpartum visit (PPV) attendance within 12 weeks of delivery increased substantially during the COVID-19 pandemic and remained elevated in the late and post-pandemic periods compared with the pre-COVID baseline. These associations persisted after adjustment for demographic, obstetric, and clinical characteristics. Additionally, Emergency room utilization within 12 weeks postpartum was reduced during early and late pandemic periods, and hospital readmissions were largely unchanged during the acute pandemic but modestly increased post-COVID.

### Results in Context:

Prior to the COVID-19 pandemic, postpartum visit attendance in the United States was variable and frequently suboptimal, with a significant proportion of individuals not completing recommended follow-up care after delivery.^1–3^ National surveillance data indicate that postpartum visit attendance declined early in the COVID-19 pandemic, with the greatest drops observed among non-Hispanic Black individuals, younger patients, and those without postpartum insurance, suggesting that the pandemic initially exacerbated existing disparities in care utilization.^16^

These findings contrast with our results, which show markedly higher attendance during all pandemic phases in our health system. Differences may reflect variation in care delivery adaptations across settings, as telehealth-based obstetric care has been shown to produce outcomes comparable to in-person care in selected settings, raising the possibility that pandemic-related innovations could have improved access to follow-up for some patients.^9,17^. For example, one study demonstrated increased odds of postpartum visit attendance and completion of postpartum depression screening during the pandemic after telehealth integration.^18^ Moreover, implementation of telehealth during the pandemic has been associated with reductions in racial disparities in postpartum care engagement, suggesting that flexible modalities may help mitigate traditional barriers to access such as transportation and childcare.^3, 5, 19–20^ Although we were unable to differentiate between telehealth and in-person visits in this study, we know telehealth was implemented in our health system during this timeframe. Therefore, our findings may reflect the impact of telehealth and outreach strategies in a predominantly Black and Hispanic population, highlighting the potential for telehealth to support postpartum engagement in historically under-resourced communities.

Additionally, emergency room utilization declined during the early and late pandemic periods, while hospital readmission rates remained largely stable during the acute pandemic and increased modestly post-COVID. The reduction in emergency room visits may partly reflect broader declines in emergency department utilization reported during the COVID-19 pandemic, particularly for non-urgent conditions, rather than true reductions in morbidity.^21^ In contrast, the stability of hospital readmissions may suggest that evolving healthcare delivery models, such as telehealth, were implemented without compromising access to necessary hospital-based care. These observations align with prior work demonstrating that changes in postpartum hospital practices during the pandemic, including shorter postpartum stays, did not lead to increased readmissions, supporting the notion that care delivery adaptations can be safely implemented while maintaining maternal safety.^22^

### Clinical Implications:

Overall, our findings demonstrate the postpartum visit attendance increased and remained higher during and after the COVID-19 pandemic. In our predominantly Black and Hispanic cohort, higher attendance during pandemic periods may reflect both increased patient motivation and health system adaptations, such as virtual visits. These strategies may help mitigate traditional disparities and provide opportunities for earlier identification and management of postpartum complications, including hypertensive disorders, gestational diabetes, and mental health conditions.

Importantly, while increased visit attendance is encouraging, it does not guarantee comprehensive or guideline-concordant care. Ensuring that each postpartum encounter includes risk-appropriate screenings, counseling, and linkage to primary or specialty care is essential to improve long-term maternal health outcomes. Our data also highlight patient subgroups, such as individuals with multiple gestations or gestational diabetes, who remain at risk for lower follow-up, emphasizing the need for tailored interventions to support these groups.

### Research Implications:

Future studies should evaluate the role of virtual care in postpartum follow-up, including which components of postpartum care can be effectively delivered through telehealth and whether virtual visits achieve clinical outcomes comparable to in-person care. In our study, we were unable to differentiate between telehealth and in-person visits, and we do not know whether visits were comprehensive postpartum encounters or focused on a single issue such as blood pressure checks or incision assessments. Understanding the content, quality, and timing of postpartum visits delivered through different modalities will be critical to determine whether increased visit attendance translates into improved maternal outcomes.

Additionally, given the sustained changes in care delivery since 2022, further research is needed to determine whether the increased postpartum engagement observed during the pandemic persists in the post-pandemic era. Longitudinal studies linking attendance and visit quality to maternal morbidity, as well as cardiovascular, metabolic, and mental health outcomes, will clarify the broader impact of evolving postpartum care models.

### Strengths & Limitations:

Strengths of this study include its large, diverse, urban cohort, the use of a comprehensive EMR-linked database with manual validation of key clinical variables, and detailed characterization of both maternal and infant outcomes. Stratification by pandemic period and adjustment for demographic, clinical, and prenatal care factors strengthen the validity of the findings. This is among the largest analyses of postpartum follow-up patterns during the COVID-19 pandemic that spans past 2021, with robust adjustment for confounders and evaluation of multiple maternal and delivery factors.

Limitations include the retrospective study design, which is subject to potential inaccuracies in EMR documentation and unmeasured factors such as social support or transportation barriers. We measured postpartum visit attendance but not the quality or comprehensiveness of care, leaving uncertainty about whether increased engagement translates into optimal long-term outcomes. The single-institution setting may limit generalizability, and while telehealth visits were increasingly integrated, our study cannot fully assess their differential impact on care quality or disparities.

## Conclusion

In this large, diverse, urban cohort, postpartum visit attendance increased substantially during the COVID-19 pandemic and remained elevated in subsequent periods, coinciding with the implementation of telehealth and sustained virtual care models within our health system. Emergency room utilization decreased during the height of the pandemic, while hospital readmissions were largely unchanged. These findings suggest that postpartum care engagement and necessary hospital-based postpartum care were maintained despite substantial changes in healthcare delivery during this period.

These results underscore the potential of flexible care delivery models, including telehealth, to enhance postpartum engagement, particularly in historically under-resourced populations. However, we cannot determine from our data whether increased attendance was driven by telehealth, in-person visits, or the content and quality of care delivered.

## Supplementary Material

This is a list of supplementary files associated with this preprint. Click to download.

- AppendixACOVID.docx

## Figures and Tables

**Table 1:** Baseline Demographics by COVID-19 Time-Period

Maternal Age, years, (mean ± standard deviation)	Pre-Covid (n = 6989)	Early COVID (n = 944)	Late COVID (n = 3273)	Post-COVID (n = 2668)	P value
	34.5 (5.9)	33.8 (5.9)	32.9 (5.9)	32.2 (6.2)	< 0.001
Infant length of stay, days (median, IQR)*	2 (2–3)	2 (2–3)	2 (2–3)	2 (2–3)	< 0.001
Number of prenatal visits (median, IQR)	12 (9–14)	12 (9–15)	13 (9–16)	13 (9–17)	< 0.001
Gestational age at first visit, weeks (median, IQR)	13.6 (10.3–19.4)	12.7 (10.1–18.3)	13.3 (9.9–21)	13.9 (10.4–21.9)	< 0.001
Gestational age at delivery, weeks (median, IQR)†	39.1 (38.3–40.1)	39.1 (38.1–40)	39.1 (38.1–39.9)	39.1 (38-39.9)	< 0.001
**Frequ**									
Race, n(%)					0.006
White	462 (6.6%)	57 (6.0%)	221 (6.8%)	156 (5.9%)	
Black	2119 (30.3%)	305 (32.3%)	971 (29.7%)	792 (29.7%)	
Asian	410 (5.9%)	52 (5.5%)	181 (5.5%)	190 (7.1%)	
Native American	30 (0.4%)	4 (0.4%)	10 (0.3%)	7 (0.3%)	
Pacific Islander/Hawiian	25 (0.4%)	6 (0.6%)	12 (0.4%)	16 (0.6%)	
Other	3208 (45.9%)	439 (46.5%)	1610 (49.2%)	1255 (47.0%)	
Mixed	49 (0.7%)	7 (0.7%)	28 (0.9%)	17 (0.6%)	
Declined/Unknown	686 (9.8%)	74 (7.8%)	240 (7.3%)	235 (8.8%)	
Ethnicity, n(%)					< 0.001
Non-Hispanic	3244 (46.4)	409 (43.3)	1410 (43.1)	1160 (43.5)	
Hispanic	3062 (43.8)	462 (48.9)	1621 (49.5)	1293 (48.5)	
Declined/Unknown	683 (9.8)	73 (7.7)	242 (7.4)	215 (8.1)	
Insurance, n(%)‡					0.016
Medicaid	5061 (76.2)	712 (77.7)	2473 (78.3)	2056 (79.2)	
Medicare	17 (0.3)	0 (0.0)	4 (0.1)	5 (0.2)	
Private Insurance	1567 (23.6)	204 (22.3)	681 (21.6)	534 (20.6)	
Parity, n(%)§					< 0.001
0	2315 (33.1)	285 (30.2)	1118 (34.2)	1017 (38.1)	
> 1	4672 (66.9)	658 (69.8)	2153 (65.8)	1650 (61.9)	
Preterm delivery, n(%)†	601 (8.6)	94 (10.0)	317 (9.7)	290 (10.9)	0.005
Mode of delivery, n(%)					< 0.001
Vaginal	4651 (66.6)	609 (64.5)	2040 (62.3)	1602 (60.0)	
Cesarean	2338 (33.5)	335 (35.5)	1233 (37.7)	1066 (40.0)	
Multi-gestation, n(%)					0.387
1	6862 (98.2)	926 (98.1)	3226 (98.6)	2613 (97.9)	
2	122 (1.8)	18 (1.9)	47 (1.4)	54 (2.0)	
3	5 (0.1)	0 (0.0)	0 (0.0)	1 (0.0)	
Diabetes, n(%)					< 0.001
Gestational	667 (9.5)	108 (11.4)	539 (16.5)	462 (17.3)	
Pre-gestational	194 (2.8)	25 (2.7)	76 (2.3)	79 (3.0)	
None	6128 (87.7)	811 (85.9)	2658 (81.2)	2127 (79.7)	
Hypertensive disorder of pregnancy, n(%)	1833 (26.2)	251 (26.6)	990 (30.3)	500 (18.7)	< 0.001
Obesity, n(%)	2109 (30.2)	341 (36.1)	1164 (35.6)	528 (19.8)	< 0.001
Prior Mental Health Condition, n(%)	692 (9.9)	94 (10.0)	360 (11.0)	197 (7.4)	< 0.001
NICU Admission, n(%)‖	736 (10.6)	97 (10.3)	317 (9.7)	286 (10.7)	0.524
Intrauterine Fetal Demise, n(%)	16 (0.2)	8 (0.9)	10 (0.3)	13 (0.5)	0.009

* For infant length of stay, n = 6974, 937, 3266, and 2695 for pre-, early, late, and post COVID respectively

† For gestational age at delivery and preterm delivery, n = 6986, 940, 3267, and 2665 for pre-, early, late, and post COVID respectively

‡ For insurance, n = 6645, 916, 3158, and 2595 for pre-, early, late, and post COVID respectively

§ For parity, n = 6987, 943, 3271, and 2667 for pre-, early, late, and post COVID respectively

‖ For NICU admission n = 6974, 942, 3269, and 2663 for pre-, early, late, and post COVID respectively

**Table 2:** Postpartum Visit (PPV) Attendance by COVID-19 Time-Period

PPV within 12 weeks, n(%)	Pre-Covid (n = 6989)	Early COVID (n = 944)	Late COVID (n = 3273)	Post-COVID (n = 2668)	P value
	3228 (46.2)	694 (73.5)	2087 (63.7)	1594 (59.8)	< 0.001
PPV within 0–3 weeks, n(%)	1705 (24.4)	452 (47.9)	1574 (48.1)	1162 (43.6)	< 0.001
PPV within 3–8 weeks, n(%)	1519 (21.7)	443 (46.9)	858 (26.2)	640 (24.0)	< 0.001
PPV within 9–12 weeks, n(%)	1220 (17.5)	208 (22.0)	636 (19.4)	525 (19.7)	0.001

**Table 3:** Unadjusted Logistic Regression Model

COVID-19 Time-Period	Unadjusted Odds Ratio	95% Confidence Interval	p-value
Pre (reference)	1.00		
Early	3.23	2.78–3.77	< 0.001
Late	2.05	1.88–2.23	< 0.001
Post	1.73	1.58–1.89	< 0.001

**Table 4:** Adjusted Logistic Regression Model

Variables	Adjusted odds ratio	95% Confidence Interval	P-value
COVID-19 Time-Period			
Pre (reference)	1.00	-	-
Early	3.50	2.97–4.11	< 0.001
Late	1.93	1.75–2.12	< 0.001
Post	1.56	1.41–1.73	< 0.001
Age (years)	1.01	1.01–1.02	0.001
Infant length of stay (days)	1.01	1.00–1.01	0.005
# Prenatal visits	1.12	1.11–1.13	< 0.001
Gestation age at first visit	1.01	1.01–1.02	< 0.001
Race			
Other (reference)	1.00	–	–
White	0.94	0.80–1.10	0.418
Black	1.21	1.08–1.37	0.001
Asian	1.02	0.85–1.23	0.825
Native American	1.27	0.66–2.41	0.475
Pacific Islander/Hawiian	2.00	1.09–3.69	0.026
Mixed	0.80	0.52–1.22	0.296
Declined/Unknown	0.94	0.79–1.12	0.517
Ethnicity			
Non-Hispanic (reference)	1.00	–	–
Hispanic	1.11	0.99–1.24	0.064
Declined/Unknown	0.99	0.83–1.19	0.916
Insurance			
Private Insurance (reference)	1.00	–	–
Medicaid	1.13	1.03–1.24	0.007
Medicare	4.22	1.38–12.95	0.012
Nulliparity	1.18	1.09–1.29	< 0.001
Preterm delivery	1.29	1.10–1.50	0.001
Cesarean Section	1.84	1.70-2.00	< 0.001
Multi-gestation			
1 (reference)	1.00	–	–
2	0.28	0.20–0.40	< 0.001
3	0.03	0.00-0.23	0.001
Diabetes			
None (reference)	1.00	–	–
Gestational	0.79	0.70–0.89	< 0.001
Pre-gestational	0.87	0.67–1.12	0.288
Hypertensive disorder of pregnancy	1.67	1.53–1.83	< 0.001
Obesity	0.96	0.88–1.04	0.347
Prior Mental Health Condition	1.48	1.30–1.70	< 0.001
NICU admission	0.96	0.82–1.11	0.563
Intrauterine Fetal Demise	0.27	0.07–1.05	0.059

**Table 5:** Secondary outcomes, ER visits and hospital readmissions

	ER Visits			Hospital Readmissions		
	Adjusted odds ratio	95% Confidence Interval	P-value	Adjusted odds ratio	95% Confidence Interval	P-value
COVID-19 Time-Period						
Pre (reference)	1.00	–	–	1.00	–	–
Early	0.61	0.49–0.76	< 0.001	1.31	0.95–1.81	0.100
Late	0.87	0.77–0.99	0.028	1.17	0.95–1.45	0.143
Post	0.91	0.80–1.04	0.177	1.35	1.07–1.69	0.010

## Data Availability

The datasets generated and analyzed during the current study are available from the corresponding author on reasonable request.

## Abbreviations

PPV: Postpartum visit
MMC: Montefiore Medical Center
EMR: electronic medical record
